# Activation of Rac-1 and RhoA Contributes to Podocyte Injury in Chronic Kidney Disease

**DOI:** 10.1371/journal.pone.0080328

**Published:** 2013-11-07

**Authors:** Andrea Babelova, Felix Jansen, Kerstin Sander, Matthias Löhn, Liliana Schäfer, Christian Fork, Hartmut Ruetten, Oliver Plettenburg, Holger Stark, Christoph Daniel, Kerstin Amann, Hermann Pavenstädt, Oliver Jung, Ralf P. Brandes

**Affiliations:** 1 Physiology I, Goethe-University, Frankfurt am Main, Germany; 2 Institute for Pharmaceutical Chemistry, Goethe-University, Frankfurt am Main, Germany; 3 Sanofi Deutschland GmbH, Frankfurt am Main, Germany; 4 General Pharmacology and Toxicology, Goethe-University, Frankfurt am Main, Germany; 5 Department of Pathology, Nephropathology, University of Erlangen-Nürnberg, Erlangen, Germany; 6 Department of Internal Medicine D, University Hospital Münster, Münster, Germany; 7 Internal Medicine/Nephrology, Goethe-University, Frankfurt am Main, Germany; Fondazione IRCCS Ospedale Maggiore Policlinico & Fondazione D’Amico per la Ricerca sulle Malattie Renali, Italy

## Abstract

Rho-family GTPases like RhoA and Rac-1 are potent regulators of cellular signaling that control gene expression, migration and inflammation. Activation of Rho-GTPases has been linked to podocyte dysfunction, a feature of chronic kidney diseases (CKD). We investigated the effect of Rac-1 and Rho kinase (ROCK) inhibition on progressive renal failure in mice and studied the underlying mechanisms in podocytes. SV129 mice were subjected to 5/6-nephrectomy which resulted in arterial hypertension and albuminuria. Subgroups of animals were treated with the Rac-1 inhibitor EHT1846, the ROCK inhibitor SAR407899 and the ACE inhibitor Ramipril. Only Ramipril reduced hypertension. In contrast, all inhibitors markedly attenuated albumin excretion as well as glomerular and tubulo-interstitial damage. The combination of SAR407899 and Ramipril was more effective in preventing albuminuria than Ramipril alone. To study the involved mechanisms, podocytes were cultured from SV129 mice and exposed to static stretch in the Flexcell device. This activated RhoA and Rac-1 and led via TGFβ to apoptosis and a switch of the cells into a more mesenchymal phenotype, as evident from loss of WT-1 and nephrin and induction of α-SMA and fibronectin expression. Rac-1 and ROCK inhibition as well as blockade of TGFβ dramatically attenuated all these responses. This suggests that Rac-1 and RhoA are mediators of podocyte dysfunction in CKD. Inhibition of Rho-GTPases may be a novel approach for the treatment of CKD.

## Introduction

Chronic renal failure is a self-perpetuating process of different etiology, which may ultimately lead to end-stage renal failure and renal replacement therapy. Numerous conditions such as diabetes mellitus, hypertension, glomerulonephritis and preexistent structural renal abnormalities can initiate chronic kidney disease. However, once the disease has become established it tends to progress to terminal renal failure even if the condition that initiated the process was successfully treated [1,2]. 

The mechanisms operative in progressive chronic renal failure are incompletely understood and numerous processes are considered relevant to mediate the different aspects of the disease. Glomerula damage is frequently observed which involves podocyte loss, proliferation of mesangial cells and thickening of the basal lamina. Also tubulo-interstitial fibrosis is commonly present with increased inflammatory activation of the renal tissue and deposition of matrix. Ultimately, these processes result in loss of active nephrons so that the remaining nephrons including their glomeruli undergo compensatory hypertrophy [2,3]. 

Interestingly, the process inducing the compensatory hypertrophy of glomerula and even the whole kidney are not well understood. Humoral factors like neuropeptide Y, vasoactive peptides or lipids that accumulate in the blood are discussed but also hydrostatic effects are considered being of relevance. Indeed, it is widely believed that chronic kidney disease induces glomerula hypertension which then further progresses renal disease [3]. 

The transglomerular pressure gradient of the healthy kidney is 30-40 mmHg and may exceed 60 mmHg in the diseased kidney [3]. Obviously, such a pronounced increase in hydrostatic pressure results in a significant increase in circumferential cellular strain [4]. This increased mechanical stress induces cellular activation and podocyte injury resulting again in the final common pathway of end-stage renal failure [5]. Podocytes as specialized epithelial cells attached to the glomerular basement membrane (GBM) are an essential part of the glomerular filter barrier preventing the loss of serum proteins into urine. Glomerulosclerosis associated with massive proteinuria is closely related to specific structural changes in podocyte complex architecture [6]. Notably, even small rearrangements of actin cytoskeleton result in effacement and disappearance of podocyte actin-rich foot processes [7]. These events represent early manifestations of progressive podocyte damage associated with detachment of podocytes from GBM and their irreversible loss. Depletion of podocytes goes in line with a robust increase in apoptosis due to activation of TGFβ signaling pathway [8]. TGFβ secreted by mesangial cells and potentially by podocytes themselves binds to its receptors on podocyte surface and initiates impairment of podocyte adhesion [9]. Another mechanism contributing to the reduction in podocyte number is activation of local tissue angiotensin system in podocytes in response to mechanical stress [10]. The increased capillary wall tension is transmitted to podocytes through cell-matrix contacts. Specific components of GBM are therefore of great importance for proper interaction with adhesion receptors localized on podocyte foot processes [11]. On the other hand, variations in GBM protein composition or their presence in the soluble form might affect cellular response to stretch [12]. 

Rho family small GTPases are known powerful regulators of actin cytoskeletal dynamics, cell adhesive interactions, motility, or cell polarity [13]. However, small GTPases also control other important cellular functions such as gene expression, proliferation, cell adhesion, and apoptosis [14]. Although Rac-1 and RhoA are needed for normal cellular function, over-activation of the proteins for example in response to angiotensin II (AngII) contributes to disease processes in the vascular but also renal system. Interestingly, HMG-CoA-reductase inhibitors (statin), if given at high concentrations, may in part inhibit Rac-1 and RhoA activity [15]. Statins decrease the formation of isoprenoids, intermediates of the cholesterol de novo synthesis, and isoprenoids are required to tether Rac-1 and RhoA to the plasma membrane. Some evidence has been presented that statins delay the progression of chronic renal failure but it is unknown whether this is due to an inhibition of small GTPases [16]. Moreover, it is not known whether Rac-1, which activates the NADPH oxidase and elicits oxidative stress, or RhoA, which for example decreases NO synthase expression and induces inflammation is the main driver of GTPase-dependent progression of renal disease [15]. 

On such basis, we determined the impact of inhibitors of Rac-1 and of RhoA-dependent kinase (ROCK), the most important downstream kinase of RhoA, in a murine model of chronic progressive renal failure. We compared their effectiveness to statins and the gold standard of treatment, ACE inhibition. Further, we analyzed the contribution of both small GTPases to podocyte function focusing on effects of mechanical stress. 

## Results

### Rac-1 and ROCK inhibition protects kidney function in vivo

**Figure 1:**
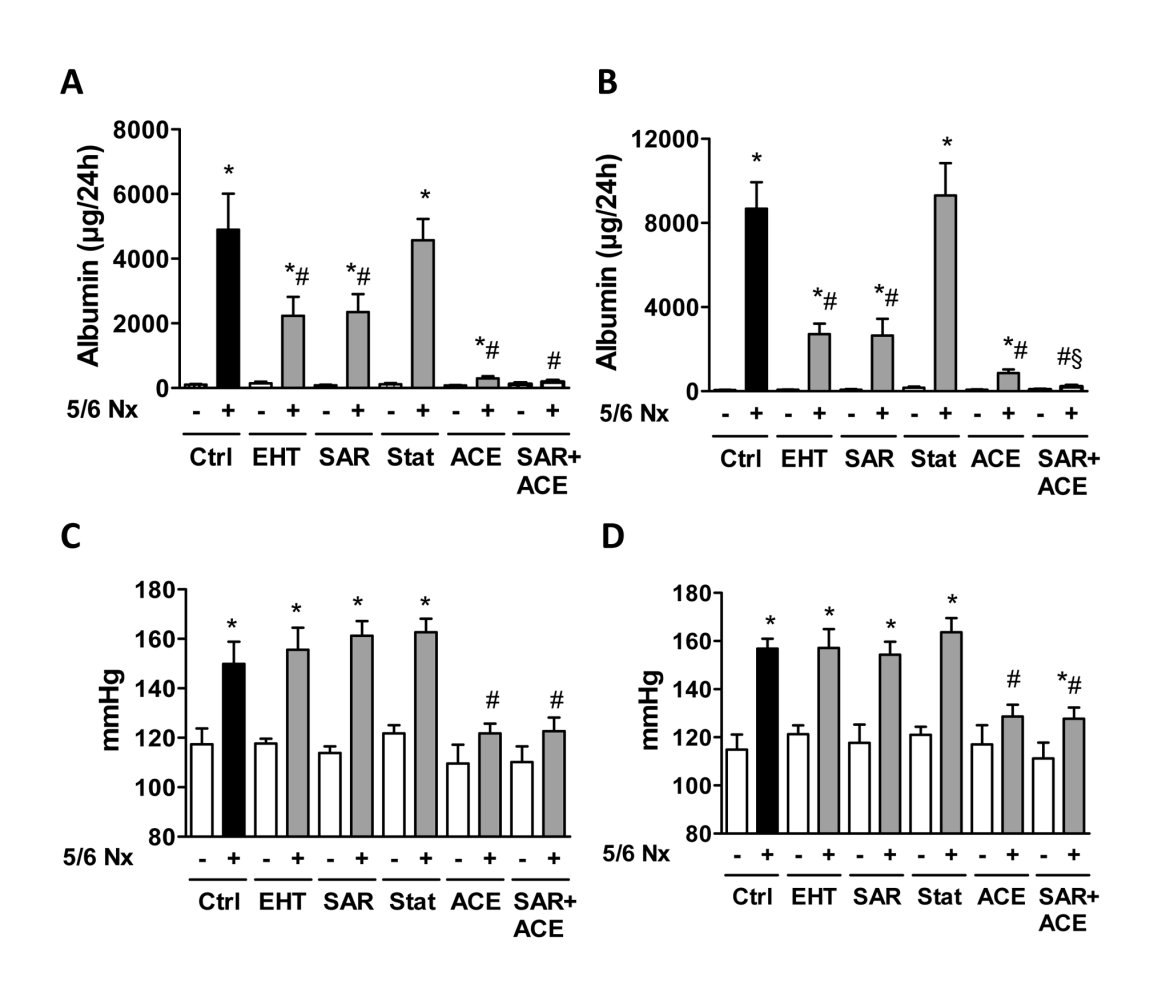
Effect of GTPase inhibition of 5/6 nephrectomy (Nx)-induced renal albumin excretion and hypertension. Albuminuria 4 weeks (**A**) and 8 weeks (**B**) and blood pressure 4 weeks (**C**) and 8 weeks (**D**) after induction of 5/6Nx. Data are means ± SEM. *P<0.05 *vs*. corresponding sham control (n=5-10 for sham and n=6-17 for 5/6Nx mice); # *P*<0.05 *vs*. 5/6Nx control (n=6-13); § *P*<0.05 *vs*. 5/6Nx + ACE (n=8-13). Ctrl: control. EHT: Rac-inhibitor EHT1864, SAR: ROCK inhibitor SAR407899, Stat: HMG-CoA reductase inhibitor Rosuvastatin, ACE: ACE inhibitor ramipril.

To investigate the contribution of the small GTPases Rac-1 and RhoA to the processes leading to progressive chronic renal failure, the 5/6Nx mice and their sham operated counterparts were treated with a set of inhibitors: the HMG-CoA reductase inhibitor Rosuvastatin, the ACE inhibitor Ramipril, the Rac-1 inhibitor EHT1864 and the ROCK (a downstream protein kinase of Rho) inhibitor SAR407899. As judged by blood urea nitrogen and plasma creatinine, 5/6Nx induced chronic renal failure, and these values were significantly lower in all groups receiving treatment (Figure **S1**). With the exception of the statin, all compounds attenuated the development of albuminuria in response to 5/6Nx at 4 weeks post op and this protective effect persisted until the end of experiment at 8 weeks. ACE inhibition was superior to Rac-1 or ROCK inhibition in preventing albuminuria but co-administration of the ROCK inhibitor to the ACE inhibitor exerted a significant add on protective effect after 8 weeks (Figure **1A&B**). 

**Figure 2:**
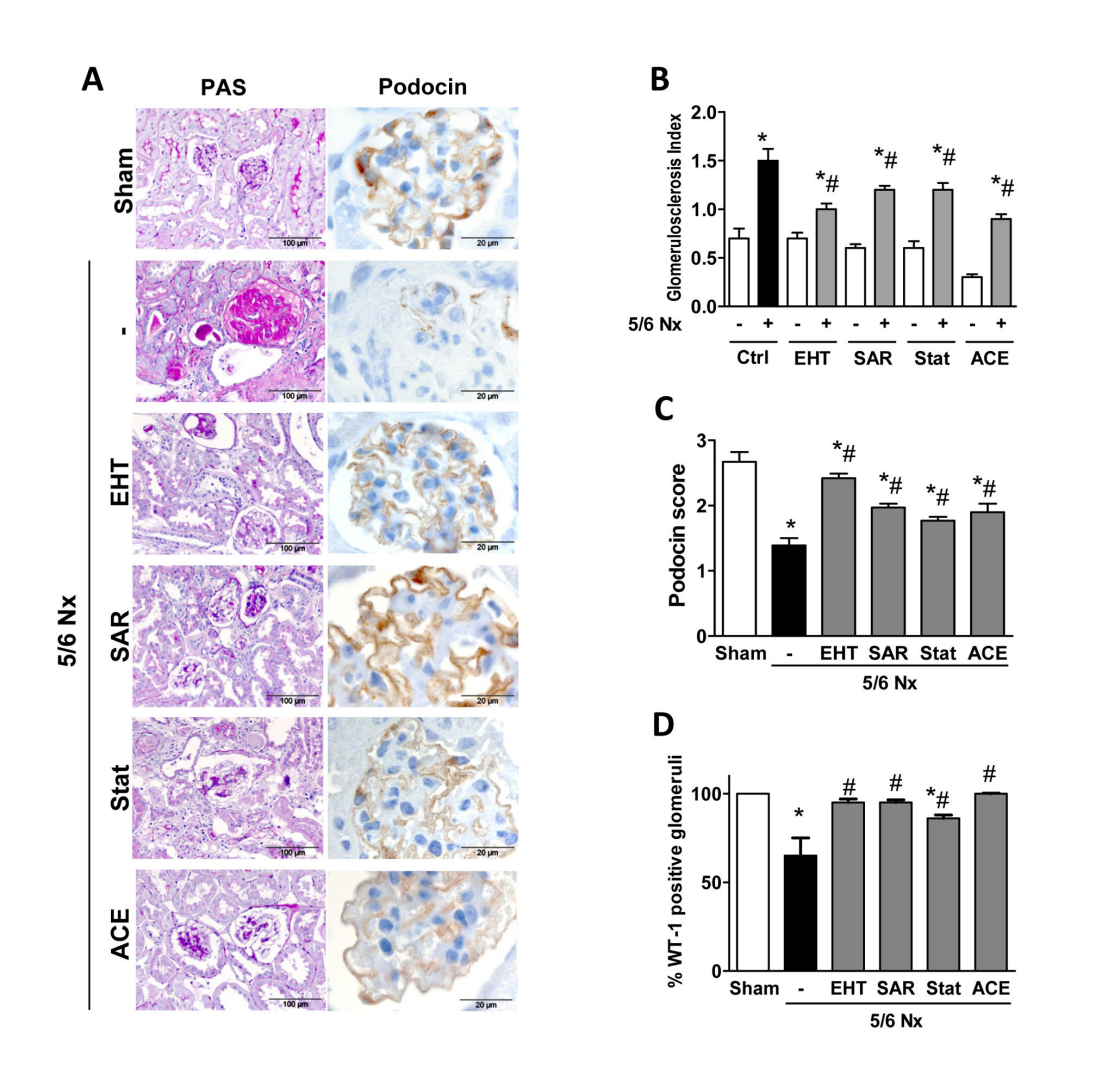
GTPase inhibition attenuates 5/6Nx-induced renal histological changes. Representative PAS (left panel; scale bar 100 µm) and podocin (right panel;, brown color, scale bar 20 µm) stained kidney sections (A), glomerulosclerosis index (**B**), podocin staining quantification (**C**), and percentage of WT-1 positive stained glomeruli (**D**) in kidneys from sham and 5/6Nx mice non-treated or treated with EHT, SAR, statin and ACE inhibitors. Data represent means ± SEM. *P<0.05 *vs*. corresponding sham control (n=5-7 for sham and n=8-18 for 5/6Nx mice); # *P*<0.05 *vs*. 5/6Nx non-treated mice (n=9-18).

Hypertension is considered a main driver of the disease process in the 5/6Nx model and indeed, mice became overtly hypertensive after renal mass reduction. As expected, ACE inhibition prevented hypertension development throughout the experiment. The other compounds, however, had no effect on blood pressure (Figure **1C&D**) suggesting that the protective effect of Rac-1 and ROCK inhibition is pressure independent and consequence of structural preservation of the glomerulum. Indeed, pronounced glomerulosclerosis was present in glomeruli of 5/6Nx mice without treatment and this was attenuated by Rac-1 and ROCK inhibition as demonstrated with the PAS staining. Also the ACE inhibitor was effective in partially maintaining glomerula architecture although we cannot discriminate whether this was consequence of lower glomerula hypertension or of the blockade of the deteriorating effects of AngII directly in the glomerulum (Figure **2A&B**). Interestingly, although incapable of preventing proteinuria, statin-treatment effectively attenuated glomerulosclerosis development, reiterating that already minor structural changes of the glomerulus are sufficient to induce gross proteinuria. The cell of glomerulus most sensitive to structural alteration is the podocyte. Staining for the specific markers podocin and WT-1 revealed that glomeruli from remnant kidneys were depleted of podocytes. Treatment with any of the compounds used in the present study prevented loss of podocyte markers (Figure **2A&C&D**), and again, the statin exerted the weakest effect (Figure **2D**).Tubular injury and renal fibrosis are often secondary to glomerula disease. Indeed, the scores for these afflictions recapitulated the glomerulosclerosis index (Figure **S2**). 

**Figure 3:**
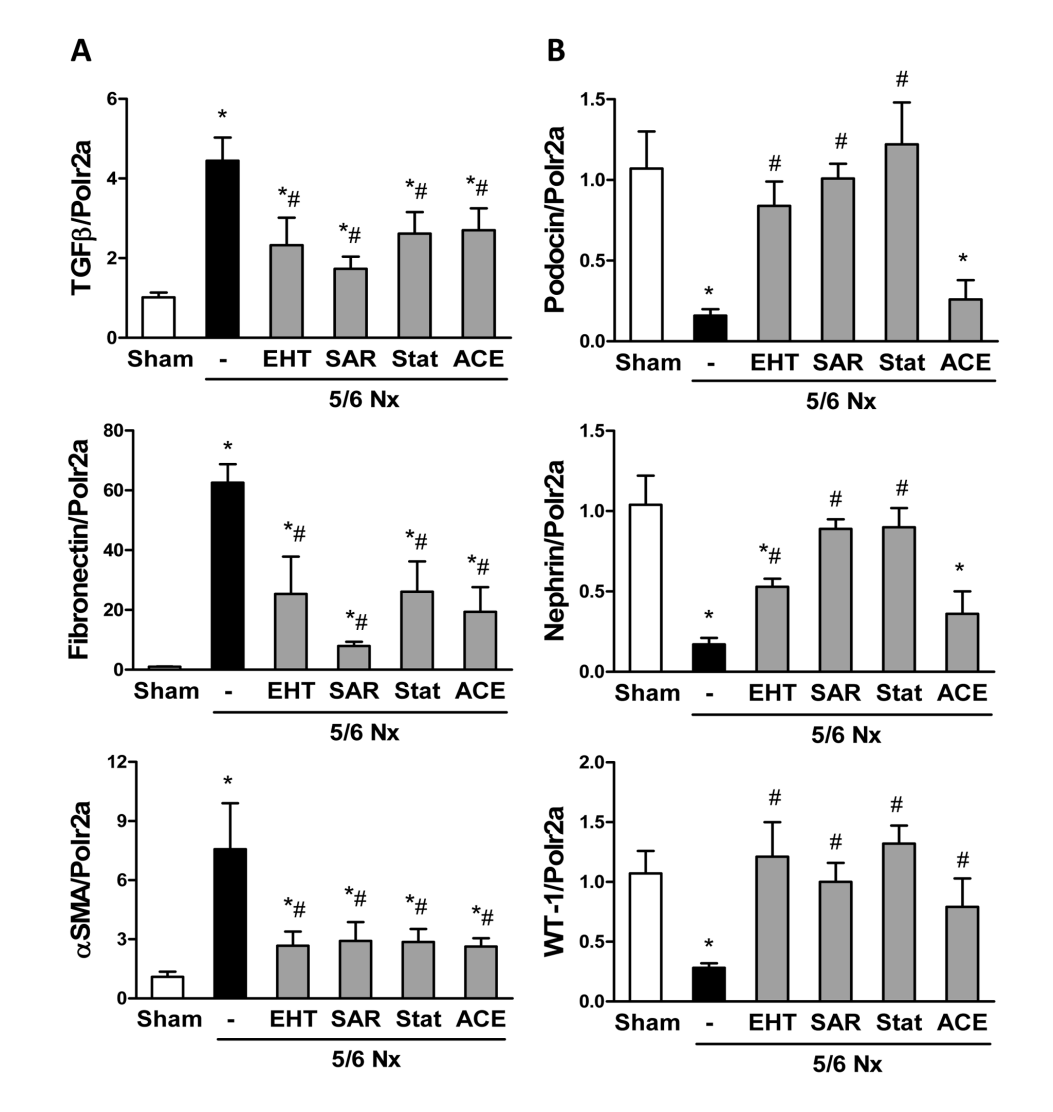
GTPase inhibition prevents 5/6Nx-induced renal fibrosis. RT-PCR analysis of renal TGFβ, fibronectin and smooth muscle α-actin (α -SMA) expression (**A**), and expression of podocyte specific markers podocin, nephrin and WT-1 (**B**) detected in renal cortex homogenates. Polymerase 2a (Polr2a) served as housekeeping gene. Data are expressed as mean ± SEM from four mice of each group. *P<0.05 *vs*. corresponding sham control; # *P*<0.05 *vs*. 5/6Nx control.

Expression pattern of TGFβ, a master profibrotic cytokine, as determined by RT-PCR from renal cortex, confirmed the histological findings. Its disease-induced overexpression was attenuated in kidneys from mice treated with any of the inhibitors applied to a similar extent (Figure **3A**). Similar patterns were observed in expression of two fibrosis indicators; fibronectin and α -SMA (Figure **3A**). These observations demonstrate that Rac-1 and ROCK inhibition reduce renal fibrosis in response to 5/6Nx. 

### Rac-1 and ROCK inhibition reduces podocyte injury in vivo

Although the disease condition studied here in particular affects glomeruli, the RT-PCR results are primarily a reflection of changes occurring in the tubulo-interstitial compartment, which is more abundant in the renal cortex. Although this aspect is a limitation of our study and studies on isolated glomeruli would have been desirable, RT-PCR for specific podocyte markers confirmed the data obtained by histology: The mRNA expression of podocin, nephrin, and WT-1 was dramatically reduced after 5/6Nx compared to WT controls. Statin treatment as well as Rac-1 or ROCK inhibition prevented the loss of podocyte marker expression in response to 5/6Nx (Figure **3B**). Interestingly, ACE inhibitor only prevented the loss of WT-1 mRNA expression, whereas the loss of nephrin and podocin mRNA expression in response to 5/6Nx was not affected by ACE inhibition. Although this finding is interesting, it might be due to secondary changes induced by the ACE inhibitor and is unlikely to indicate that the compound failed to protect podocytes. 

### Inhibition of Rac-1 and ROCK prevents stretch-induced epithelio-mesenchymal phenotype switch of cultured podocytes

**Figure 4:**
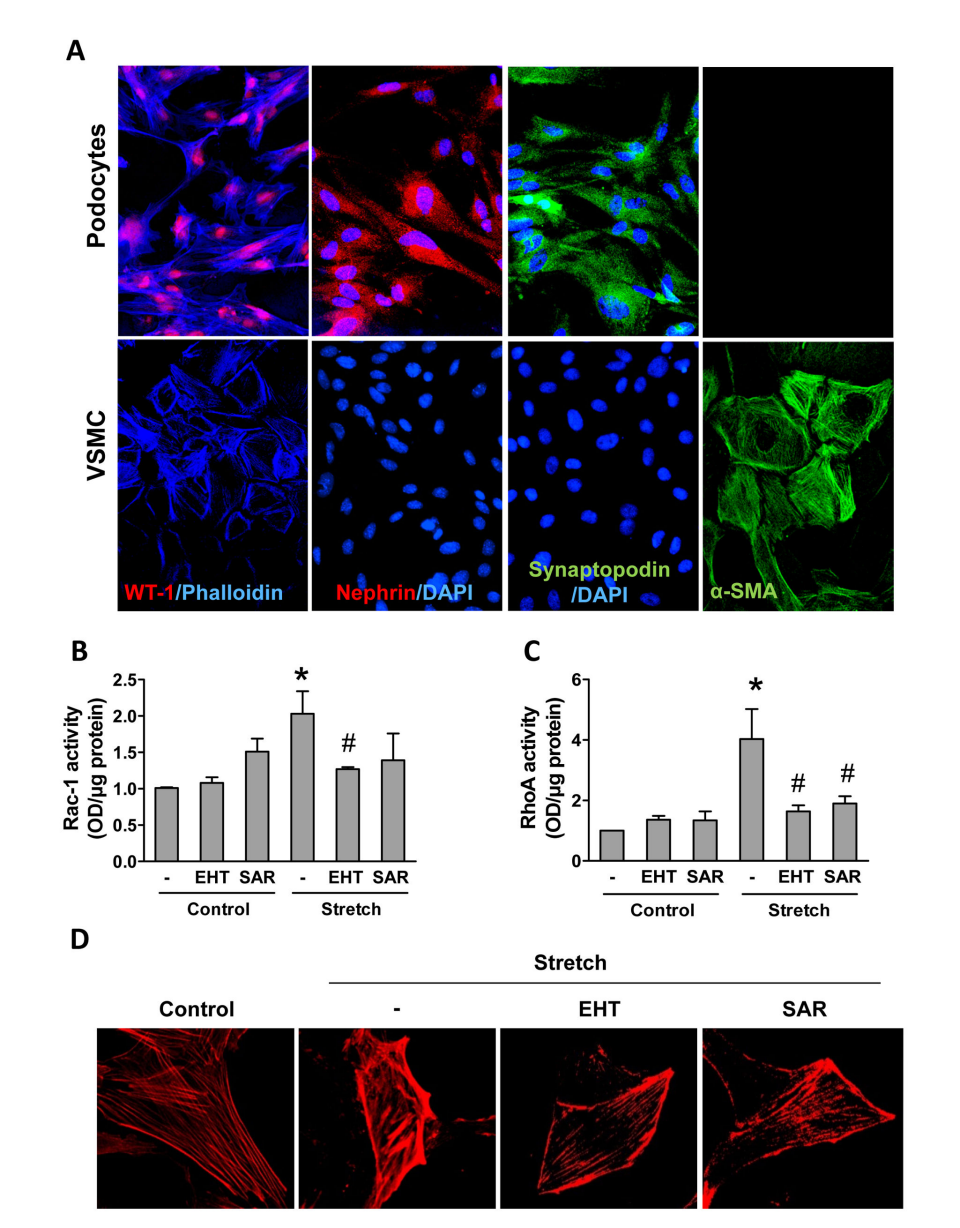
Stretch increases Rac-1 and RhoA activity in murine podocytes. Expression of the podocyte proteins WT-1, nephrin and synaptopodin as well as smooth muscle α-actin in podocytes and rat smooth-muscle cells (**A**). Activation of Rac-1 (**B**) and RhoA (**C**) in primary podocytes in response to stretch (1h) with or without preincubation with EHT (1µM) or SAR (1µM). Data are means ± SEM. *P<0.05 *vs*. corresponding non-stretched control; # *P*<0.05 *vs*. stretched control (n=3 for Rac-1 assay and n=5 for RhoA assay). Phalloidin staining of the actin cytoskeleton in podocytes after 24-hour stretch with or without EHT (1µM) or SAR (1µM) treatment (**D**).

To further investigate the mechanism underlying the protective role of Rac-1 and ROCK inhibition in chronic renal failure we performed cell culture experiments in murine podocytes. As our *in vivo* studies were based on mouse on the SV129 genetic background, we isolated glomeruli from kidneys of this strain to obtain podocyte culture. Isolated primary podocytes expressed podocyte-specific proteins like WT-1, nephrin, and synaptopodin but not the smooth-muscle cell marker α -SMA (Figure **4A**; Figure **S3**). To simulate the glomerula hypertension-induced increase in pressure, podocytes were exposed to static stretch in the Flexcell device (15% elongation). Mechanical strain increased activity of both Rac-1 and RhoA, as determined by G-LISA. Interestingly, ROCK inhibition as well as Rac-1 inhibition prevented the stretch-induced increase in GTPase activity (Figure **4B&C**). Given that ROCK is down-stream of RhoA and that EHT1864 is specific for Rac-GEFs this observation demonstrates that sensing of mechanical load leading to GTPase activation requires an active cytoskeleton. Indeed, podocytes under stretch condition underwent strong actin polymerization and this was attenuated by Rac1 and ROCK inhibition (Figure **4D**)**.**

**Figure 5:**
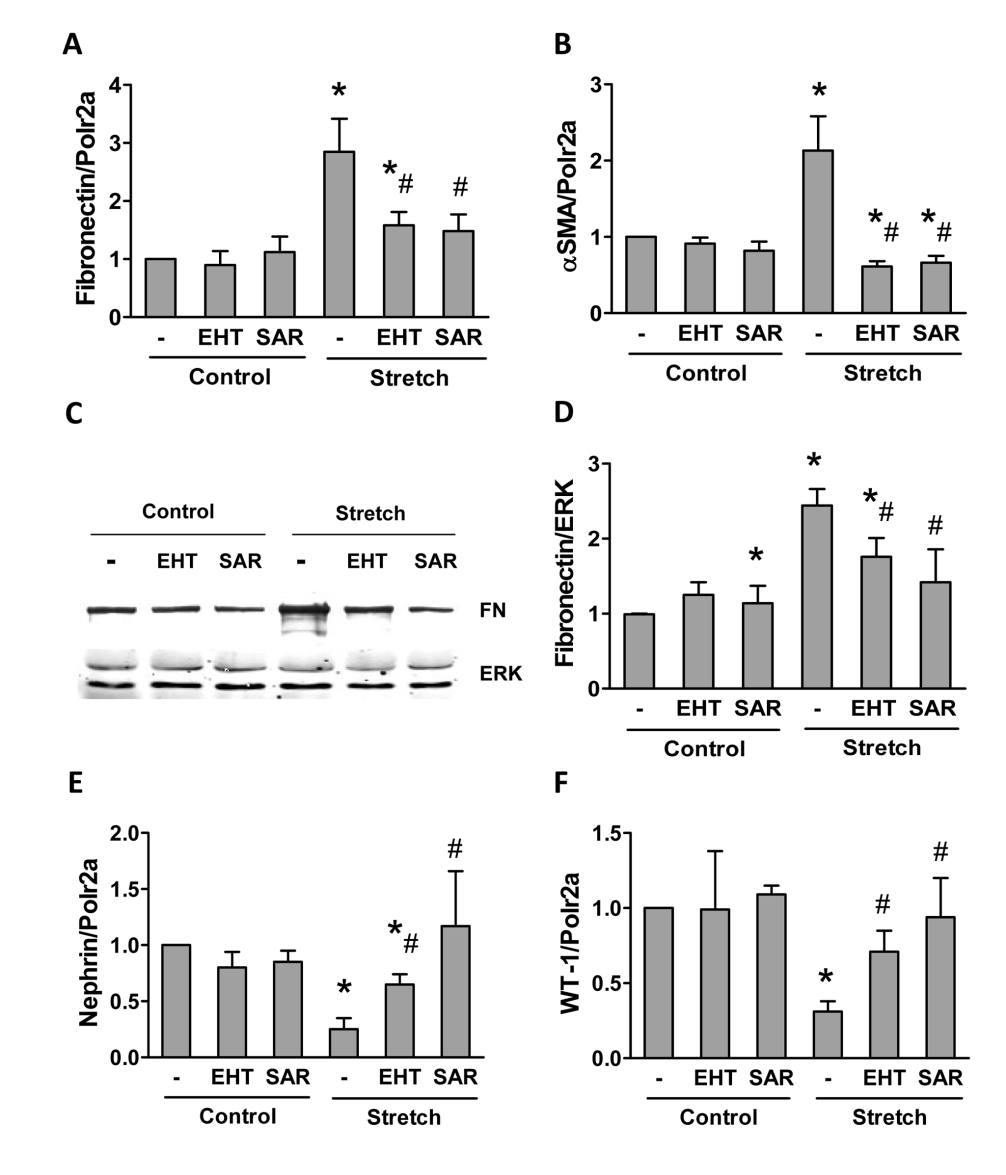
Stretch-induced signaling is sensitive to GTPase inhibition. Effect of stretch (24hours) on podocytes in the presence or absence of EHT (1µM) or SAR (1µM) on fibronectin mRNA expression (**A**), α-smooth muscle actin mRNA expression (**B**) fibronectin protein expression (**C**&**D**) and nephrin and WT-1 mRNA expression (**E**&**F**). ERK was used as reference for protein expression, polymerase 2a (Polr2a) as housekeeping gene for mRNA expression. Data represent the mean ± SEM of three independent experiments. *P<0.05 *vs*. corresponding non-stretched control; # *P*<0.05 *vs*. stretched control.

Given the activation of GTPase, we infer that stretch leads to podocyte activation and initiates podocyte dedifferentiation. Indeed, mechanical stretch enhanced the expression of fibronectin and α -SMA in podocytes and both, the induction of mRNA and protein of fibronectin and α -SMA were prevented by Rac-1 and ROCK inhibition (Figure **5A-D**). Podocyte dedifferentiation was as well accompanied by a loss of the podocyte-specific markers nephrin and WT-1 and also these changes were prevented by Rac-1 and ROCK inhibition (Figure **5E&F**). 

### TGFβ mediates stretch-induced podocyte activation

**Figure 6:**
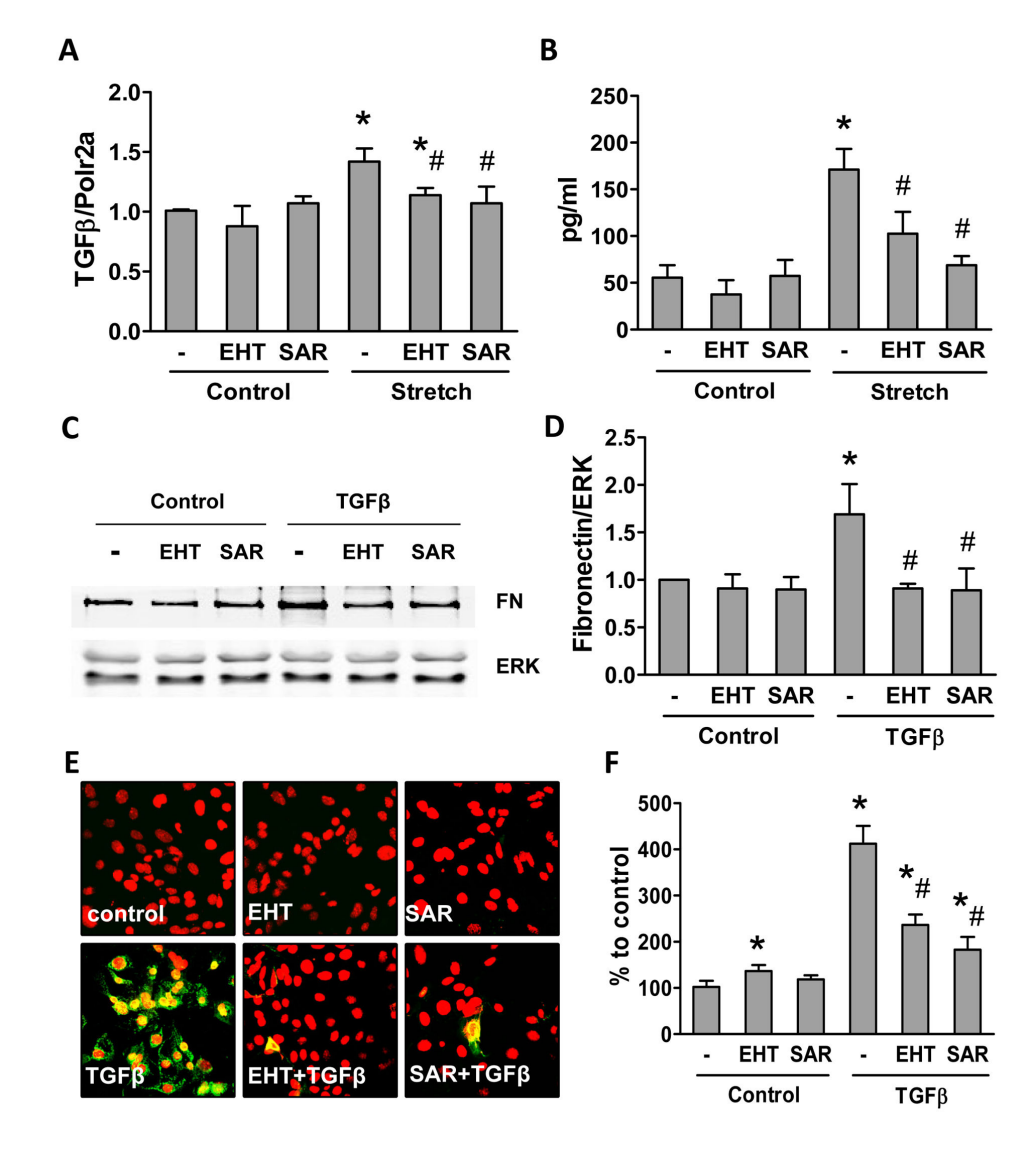
TGFβ-induced podocyte signaling involves small GTPases. RT-PCR for TGFβ (**A**) in podocytes with and without exposure to stretch (24hours) in the absence or presence EHT (1µM) or SAR (1µM). (**B**) TGFβ protein expression detected by ELISA in podocytes with and without exposure to stretch (24hours) and treated with or without EHT (1µM) or SAR (1µM). Western blot for fibronectin (FN) (**C**) and quantification (**D**) produced by primary podocytes stimulated with or without TGFβ (10 ng/ml), in the absence or presence of EHT (1µM) or SAR (1µM). Annexin V staining after exposure with or without TGFβ (24h) (**E**) and its quantification (**F**) in podocytes treated with or without EHT (1µM) or SAR (1µM). Data represent the mean ± SEM of three independent experiments. *P<0.05 *vs*. corresponding non-stretched control (A, B) or non-stimulated control (D, F); # *P*<0.05 *vs*. stretched control (A, B) or TGFβ stimulated control (D, F).

**Figure 7:**
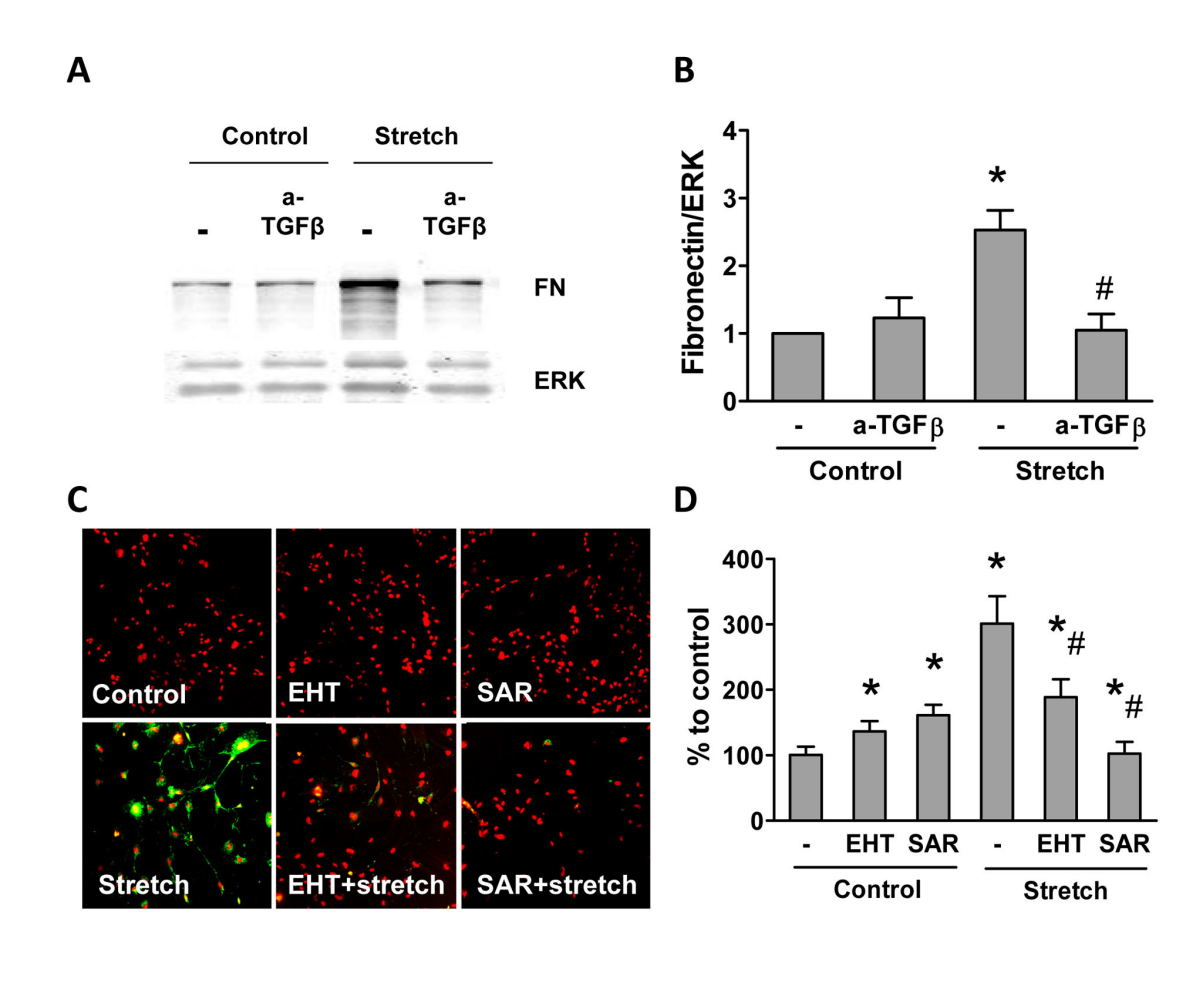
TGFβ mediates stretch-induced signaling in podocytes. Western blot for fibronectin (FN) (**A**) and quantification (**B**) of primary podocytes with and without stretch exposure in the absence or presence of anti-TGFβ neutralizing antibody (40 µg/ml). Annexin V activation (**G**) and its quantification (**H**) in podocytes with and without stretch-exposure treated with or without EHT (1µM) or SAR (1µM). The data shown are representative of three independent experiments and are means ± SEM (B, D). *P<0.05 *vs*. corresponding non-stretched control; # *P*<0.05 *vs*. stretched control.

One of the strongest cytokines promoting fibrotic processes is TGFβ. TGFβ stimulates matrix production, induces epithelial cell apoptosis and induces epithelio-mesenchymal phenotype switch. On such a basis we determined a potential involvement of TGFβ in stretch-induced podocyte signaling. Stretch increased TGFβ mRNA and protein expression in podocytes via a pathway sensitive to Rac1 and ROCK inhibition (Figure **6A&B**). Also down-stream TGFβ signaling required the small GTPases. Recombinant TGFβ induced fibronectin expression (Figure **6C&D**) and podocyte apoptosis (Figure **6E&F**) and also these processes were sensitive to the inhibition of Rac1 and ROCK. We therefore hypothesized that also the stretch-mediated induction of fibronectin may involve TGFβ. Indeed, application of a TGFβ neutralizing antibody prevented stretch-induced fibronectin deposition (Figure **7A&B**). In line with this, prolonged stretch induced podocyte apoptosis and also this could be blocked by the inhibition of Rac1 and RhoA (Figure **7C&D**).

## Discussion

In the present study we determined the impact of Rac-1 and ROCK inhibition on the progression of chronic renal failure in mice and on stretch-induced phenotype switch in cultured murine podocytes. In vivo, inhibition of Rac-1 and ROCK attenuated structural remodeling and albuminuria in a pressure-independent manner. In cultured podocytes subjected to static strain, small GTPases were identified as important mediators of podocyte activation which were involved in stretch-induced TGFβ induction and down-stream TGFβ signaling. 

Increased glomerular pressure is an important factor known to cause podocyte injury [17]. Intraglomerular hypertension and hyperfiltration develop during chronic renal failure, in part as a consequence of altered autoregulation of the glomerular microcirculation involving vasodilatation of afferent and efferent arteriole [18]. Current clinical strategies in slowing the progression of chronic kidney disease mainly involve ACE inhibitors and AT1 receptor blockers [19]. These prevent glomerular hypertension by a vasodilator effect on the efferent arteriole. Moreover, ACE inhibitors attenuate the inflammatory response during chronic renal failure by preventing local AngII production. By this mechanism they mitigate structural changes and attenuate dedifferentiation [20]. In the present study we were able to confirm the reno-protective effect of ACE inhibition in the 5/6Nx model and also observed a pronounced anti-hypertensive effect of the compound. Nevertheless, we did not measure intraglomerular pressure, which represents a major limitation of the present study as systemic blood pressure is not an indicator of glomerular hypertension. With the exception of the ACE inhibitor, none of the compounds affected systemic blood pressure, but they still might have lowered intraglomerular pressure and by that aspect delayed disease progression. 

Although ACE inhibitors are successful in delaying the progression of chronic renal failure, few alternative treatment options exist and thus there is great medical need for novel strategies combating this disease [2]. Modulating Rho-GTPase signaling might be one of these: RhoGTPases affect different renal cells [21-23] and their activation is thought to contribute to renal injury [24,25]. Rho kinase has been implicated in cell proliferation, migration and apoptosis as well as matrix deposition [26], which makes it an attractive candidate altering differentiating processes. ROCK inhibition with Fasudil reduced the trace albuminuria in STZ-diabetic rats [27]. Similar results were obtained in a rat model of accelerated kidney disease (combination of STZ-diabetes and unilateral nephrectomy) [28]. Importantly, prolonged treatment with Fasudil also did not lower blood pressure in rats. Similar results were obtained in spontaneously hypertensive rats subjected to 5/6Nx [29]. Also in this model, Fasudil reduced albuminuria in a blood pressure-independent manner. Despite this, Fasudil has been criticized for limited specificity towards Rho kinase [30] and thus some of the effects currently attributed to ROCK inhibition by Fasudil might be mediated by protein kinase A or protein kinase C inhibition. On such a basis, we determined whether the highly specific ROCK inhibitor SAR407899 [31] elicits protection in the murine remnant kidney model. Previous observations indicated that SAR407899 has pressure-independent effects similar as Fasudil: It reduced migration of a monocyte cell line and attenuated PDGF-induced smooth muscle cell proliferation [31]. Indeed, we here observed that also specific ROCK inhibition attenuated progression of renal failure in mice. Given that mechanistically, ACE inhibitors and ROCK inhibitors act on different pathways to prevent renal disease, we determined the impact of combined application of Ramipril and SAR407899 on albuminuria, the most sensitive parameter of glomerula dysfunction. Importantly, ROCK inhibition conferred additional protection on top of ACE inhibition. This observation is very important as it opens novel therapeutic options for patients experiencing disease progression under ACE inhibitor treatment. Additional studies therefore should be undertaken to test whether our observation can be translated into other preclinical models of chronic kidney disease.

As a second GTPase-dependent mechanism, we focused on Rac-1. This protein also belongs to the Rho family of GTPases but differs from RhoA in its down-stream targets like NADPH oxidase and p21-PAK [15]. Rac-1 is required for cell migration as it is involved in lamellipodia formation and thus inhibition of this GTPase reduces inflammatory cell accumulation. Rac-1 is also involved in NADPH oxidase activation and thus inhibition of Rac-1 reduces oxidative damage. For renal cells, it was shown that the Rac-1 inhibitor NSC23766 blocks oxalate-induced NADPH oxidase-mediated oxidative cell injury in tubular cells [32]. In vivo NSC23766 reduced the hyperinflammatory response and barrier dysfunction of the lung after LPS-treatment [33]. It also attenuated lung and gland damage in the cerulein-induced pancreatitis [34]. Interestingly, inhibition of Rac-1 by NSC23766 attenuated neuronal cell apoptosis in response to ischemia by preventing JNK activation [35] providing a potential explanation for the anti-apoptotic effect of EHT1864. 

Recently, EHT1864, the Rac-1 inhibitor also used in the present study, was tested in the angiotensin II-salt-induced kidney injury model of double transgenic Tsukuba hypertensive mice. Similarly as in the present study, EHT1864 attenutated renal injury and fibrosis without affecting blood pressure [36]. In salt-sensitive Dahl rats, also NSC23766 attenuated proteinuria by a pathway involving the mineralocorticoid receptor. As NSC23766, however, also effectively lowered the blood pressure in Dahl rats, it is unclear whether this was a simple pressure-dependent effect [37]. Both models differ considerably from the one used in the present study: Not only that they focus on a very specific disease entity, they heavily rely on oxidative stress and aldosterone and its signaling via the mineralocorticoid receptor. 

In our mechanistic analysis we focused on the aspect of podocyte injury and identified a novel role of Rac-1 in TGFβ signaling. TGFβ can activate Rac-1 in mesenchymal cells through PAK2 [38]. Although we observed that inhibition of Rac-1 blocked TGFβ-induced Rac-1 activation, the signal transduction from TGFβ to Rac-1 is unclear as the epithelial enriched proteins Erbin and Merlin usually prevent TGFβ-mediated Rac-1 activation [39]. Whether or not this pathway is operative in podocytes is not known. TGFβ is an established driver of phenotype switch [40]. In response to high glucose in immortalized mouse podocytes the process could be attenuated by siRNA directed against Rac-1 [41]. 

The activity of Rac-1 and RhoA, the upstream activator of ROCK is dependent on geranylgeranylation of the small GTPase. This tethers the protein to the membrane where it interacts with its upstream and down-stream effectors. High concentrations of statins are therefore effective in reducing Rho-GTPase activation [15]. Indeed, we observed that Rosuvastatin similar as SAR407899 and EHT1864 attenuated the structural remodeling in response to 5/6Nx. Surprisingly, Rosuvastatin had no effect on albuminuria, although the compound reduced albuminuria in the rat remnant kidney model [42]. To our knowledge, the present study is the first to explore statin treatment in the murine remnant kidney model. Thus specific conditions applying to the murine system may therefore underlie their lack of effect. The remnant kidney model is rarely used in mice as only certain strains, like SV129, develop albuminuria and progressive renal failure [43]. On the other hand, Rosuvastatin treatment was the least effective in prevention of podocyte loss in our study and left a substantial amount of podocyte-free glomeruli in the remnant kidneys. Despite this, also in another study Rosuvastatin applied to ApoE-/- mice failed to reduce albuminuria [44] but in that study, albuminuria was anyway very discrete. 

Chronic kidney disease is an exceedingly complex process which affects basically every cell in the kidney and thus a comprehensive characterization of renal changes occurring in the disease process is a demanding task. Ideally, determination of glomerula filtration rate, plasma lipids, electron microscopy and a separate analysis of the changes occurring in the glomerula and tubule fraction should be carried out in addition to the measurements performed in the present study. These important analyses, however, have not been carried out in the present study which represents major limitations. 

Of the cells contributing to chronic renal failure, podocytes recently gained a lot of interest as they form essential components of the filtration barrier and as proteinuria after barrier breakdown drives interstitial and tubular changes [40]. Numerous humoral factors, in particular angiotensin II but also NPY, contribute to podocyte activation in chronic renal failure [45] but also the mechanical environment changes. Given their exceedingly complex structure even minor changes in transglomerular hydrostatic pressure will result in activation of podocyte stretch signals. The cytoskeleton is essential for mechanosensing as the tensegrity structure of actin is required to establish points for force development and perception. As RhoGTPases control actin dynamics they are both required for mechanosensing and effectors of mechanosensing. Indeed, we observed that static strain increases RhoA and Rac-1 activity and previously it noted that stretch, albeit applied as cyclic stretch, leads to ROCK inhibitor-sensitive stress fiber formation in podocytes [46]. Stretch then leads to compensatory cell contraction followed by spreading as well as activation of gene expression. The latter aspect was evidenced by an induction of TGFβ and an increase in matrix synthesis and was also sensitive to GTPase inhibition. Previously, it was noted in conditionally immortalized mouse podocytes that stretch increases TGFβ and AT1 receptor expression and the remnant kidney model exhibited similar features as stretch in vivo [10]. Although we focused on TGFβ in the present study, an intense crosstalk to the AngII system was documented previously. For example, the stretch-induced activation of TGFβ is in part mediated by reactive oxygen species (ROS) formation stimulated by the AngII system [40]. Rac1 is required for AngII-induced ROS formation. Therefore, it is conceivable that stretch-induced matrix induction mediated through TGFβ not only depends on RhoA to stabilize that cytoskeleton but also on Rac1. 

Podocytes exposed to mechanical stretch also undergo apoptosis [10] and also TGFβ is known to induce podocyte apoptosis [8]. The degree of stretch applied in the present study was rather moderate and thus it is conceivable that not direct cell damage but stimulation of TGFβ signaling was involved. The mechanisms linking GTPases and podocyte apoptosis have not been explored. Nevertheless, numerous reports in other cell types document that inhibition of ROCK [47,48] or Rac-1 [35,49] has anti-apoptotic effects. 

In conclusion, in the present study we have demonstrated that inhibition of Rac1 as well as of ROCK is an effective novel approach to delay the progress of disease in the murine remnant kidney model. As underlying mechanism we demonstrate that the inhibitors prevent strain-induced, TGFβ-mediated apoptosis and phenotype switch of podocytes. 

## Materials and Methods

### Animals

Male SV129 mice were purchased from Harlan (Borchen, Germany). All experiments were initiated at a mouse age of 8-weeks. Animals were housed in an SPF facility with 12/12 hours day/night cycle and free access to chow and water. Body weight was monitored at least at the beginning and at the end of the experiments. 5/6 nephrectomy was performed under isoflurane / buprenorphine anesthesia as previously described with some modifications [43] and all efforts were made to minimize suffering. Briefly, left dorsal longitudinal incision was performed to expose the left kidney. The upper branch of the left renal artery was ligated by 6-0 prolene suture to produce about one third area with visible renal ischemia infarct; the lower pole of the left kidney (about one third kidney size) was removed by cautering. After 7 days of recovery, the right kidney was exposed in a similar preparation and removed after decapsulation and ligation of the vessels and the ureter to induce a total 5/6 nephrectomy . The control animals were sham operated in parallel by decapsulating the kidney. Early mortality within the first 3 days was approx. 20%. Subsequently, animals were randomized to the treatment groups. The substances were administered as follows: The statin rosuvastatin (final concentration 50 mg/L), the ACE-inhibitor ramipril (final concentration 40 mg/L) and the Rho kinase inhibitor SAR 407899 (final concentration 50 mg/L) were given with the drinking water, the Rac-1 inhibitor EHT 1864 (40 mg/kg) was given by intraperitoneal injection. SAR407899 [31] and Ramipril were provided by Sanofi (Frankfurt). EHT 1864 was synthesized by the authors according to known methods in literature [50,51]. All animal experiments were conducted in accordance with the German Animal Protection Act and were approved by the University Animal Care Committee as well as Ethics Review Committee for laboratory animals of District Government of Darmstadt, the local government of Hessen, Germany, under the approval numbers F61/16 and F28/12.

### Blood pressure measurements

Systolic blood pressure was assessed at 4 and 8 weeks after the initiation of treatment by an automated tail-cuff Blood Pressure Monitor (Visitech) in conscious, trained mice at room temperature as reported previously. On the day of the measurement, 20 recordings were registered and of them the last 10 were used for data analysis [52]. 

### Determination of plasma creatinine and urea

Plasma creatinine was determined by the HPLC system Elite La Chrome VWR. An isocratic method was used with 15 mM lithiumacetat pH 4.7 / methanol (90:10 v/v). The cation exchange column Hamilton PRP-X200 10 µm 4.1 x 150 mm and a flow rate of 0.5 ml/min was used for chromatography. Plasma samples (100 µl) were precipitated with 100 µl methanol (-20°C) and centrifuged for 10 min at 13,000 g by 4°C. Supernatant was diluted with 1 x volume of 15 mM lithiumacetat pH 4.7 and 30 µl of the diluted plasma were injected into the HPLC system. Detection was in UV mode at 234 nm. For measurement of urea level in plasma Urea Assay Kit (BioCat, Heidelberg, Germany) was used following the manufacturer’s instruction. 

### Determination of urinary albumin and creatinine

Mice were placed in metabolic cages (Tecniplast) for 24-hour urine collection. Urinary albumin excretion was determined by ELISA for mouse albumin (Bethyl Laboratories Inc., Montgomery, Texas) and urinary creatinine levels were estimated with the Creatinine assay kit (Labor-Technik, Berlin, Germany) according to the manufacturer’s instruction. 

### Morphological Studies

Kidneys were perfused with phosphate-buffered saline (PBS), subsequently fixed with 4% paraformaldehyde/PBS solution and embedded in paraffin. Renal sections (2 µm) were deparaffinized and stained with haematoxylin/eosin (HE), periodic acid Schiff (PAS) and Sirius red (fibrous tissue stain). The sections were analyzed for parameters as reported previously [53] by investigator blinded to the study. Matrix accumulation and sclerosis of the glomerular tuft (Glomerulosclerosis index) were determined in PAS and HE stained sections with the scoring system used: 0 – normal glomerulus; 1 – mesangial expansion or sclerosis involving up to 25% of the glomerular tuft; 2 – sclerosis of 25 to 50%; 3 – sclerosis of 50 to 75% and/or segmental extracapillary fibrosis or proliferation; and 4 - global sclerosis >75%, global extracapillary fibrosis or complete collapse of the glomerular tuft. Tubular atrophy, dilatation, interstitial inflammation and interstitial fibrosis (Tubulointerstitial damage index) were determined in HE sections 10 fields per kidney were randomly graded for tubulointerstitial damage at a magnification of 100x using a similar scoring system from 0-4: 0 – normal tubulointerstitial structure; 1 – lesions involving less than 25% of the area; 2 – lesions affecting 25 to 50%; 3 – lesions involving more than 50% up to 75%: and 4 – tubulointerstitial damage of almost the entire area. Sirius red stained kidney sections were graded for fibrosis at a magnification of 100x using 10 randomly selected power fields and a similar scoring system from 0-4: 0 - normal slight staining of the basement membrane; 1 - increased sirius red positive staining involving less than 25% of the tubulointerstitial area; 2 - marked sirius staining in 25 to 50%; 3 - sirius red staining involving more than 50% up to 75%: and 4 - pronounced sirius staining of almost the entire area.

### Cell culture and stimulation

Primary podocytes were isolated as described before [54]. Briefly, after transcardiac perfusion with 8 x 10^7^ inactivated Dynabeads (Dynabeads M450 tosylactivated, Dynal) suspended in HBSS medium (Invitrogen), the kidneys were minced into 1 mm^3^ pieces. The kidney pieces were digested with 1 mg/ml collagenase A (Calbiochem) in HBSS at 37°C for 30 minutes and subsequently gently pressed through a 100 µm cell strainer using a flattened pestle. The filtrate was passed through a new strainer, cell suspension was centrifuged at 200 x *g* for 5 minutes and pellet was resuspended in 5 ml of HBSS. Glomeruli containing Dynabeads were gathered by a magnetic particle concentrator, washed three times with HBSS and finally were cultured in podocyte medium consisting of 1640 RPMI supplemented with GlutaMAX (Invitrogen), 10% heat inactivated fetal calf serum (Biochrom), 100 U/ml penicillin and 100 mg/ml streptomycin, 5 mmol/L HEPES (Sigma-Aldrich), 1 g/L non-essential amino acids, 1 mmol/L sodium pyruvate (all PAA, GE healthcare), 10 mg/L insulin-transferrin-sodium selenite supplement (Roche) at 37°C. For experiments, cells were kept in serum-free medium for 24 hours and then treated with Rac-1 and ROCK inhibitors (1µM), TGFβ (10 ng/ml), or 15% static mechanical strain (Flexcell machine, Dunn) for indicated timepoints.

### Immunohistochemistry

Cells were washed with PBS and fixed with 4% PFA for 20 minutes. After incubation with primary antibodies: anti-nephrin (Acris), anti-synaptopodin, anti-WT (both Santa Cruz Biotechnology Inc.), anti-podocin, anti-a-SMA (both Sigma) or anti-phalloidin Alexa Fluor 647 conjugated (Invitrogen) appropriate secondary antibody was used: Alexa Fluor anti-rabbit 488, Alexa Fluor anti-goat 488, and Alexa Fluor anti-mouse 488 (all from Invitrogen). Nuclei were counterstained with DAPI. Staining patterns were analyzed with confocal fluorescent microscopy (LSM Zeiss 510 meta). Kidney sections were stained with anti-podocin (Sigma Aldrich) and anti-WT-1 (Santa Cruz Biotechnology Inc.). Negative controls for immunostaining included either the omission of the primary antibody or substitution of the primary antibody with equivalent concentrations of a pre-immune rabbit IgG. After antigen retrieval using a pressure cooker, primary antibodies were incubated overnight at 4°C, specific biotinylated secondary antibodies (all by Vector Lab.) were applied to tissue sections, followed by peroxidase-conjugated Avidin D (Vector Lab.), and color development with diaminobenzidine.

For each biopsy, at least 20 glomerular cross-sections for the podocin staining and 50 glomerular cross-sections for the WT-1 staining were evaluated in a blinded fashion. Podocin staining was graded semi quantitatively on a scale of 0 to 3 (0 = no staining; 1 = podocin staining is strongly reduced; 2 = podocin staining is reduced, 3 = normal podocin staining). The number of WT-1 positive cells is determined and means of WT-1 positive cells per glomerular cross-section were calculated. In addition, the percentage of glomerular cross-sections lacking WT-1 positive cells were evaluated.

### qRT-PCR

Total RNA was extracted with the RNA Mini Kit (Bio&Sell). cDNA was prepared with SuperScript III Reverse Transcriptase (Invitrogen) and random hexamer primers. Semiquantitative real-time PCR was performed with Fast Plus EvaGreen Master Mix and ROX as reference dye (Biotium) in an Mx4000 cycler (Stratagene) with primer sequences as follows: α -SMA forward primer (5'-ACA GAG GCA CCA CTG AAC CCT AAG-3') and reverse primer (5'-ACA ATC TCA CGC TCG GCA GTA GTC-3'); fibronectin forward primer (5'-ATG CAC CGA TTG TCA ACA GA-3') and reverse primer (5'-TGC CGC AAC TAC TGT GAT TC-3'); TGFβ forward primer (5'-TGA CGT CAC TGG AGT TGT ACG G-3') and reverse primer (5'-GGT TCA TGT CAT GGA TGG TGC-3'); nephrin forward primer (5'-GCA TCA CTC TGC AGG TCA CCT TTC-3') and reverse primer (5'-AGG CCA TCC ATG ACT GTC TCA TCC-3'); synaptopodin forward primer (5'-AGG GAC CAG CCA GAT AGA GCA AAG-3') and reverse primer (5'-CAT GGG ACT GCG GGA CAT TAT GTG-3'); podocin forward primer (5'-AAG TGC GGG TGA TTG CTG CAG AAG-3') and reverse primer (5'-TGT GGA CAG CGA CTG AAG AGT GTG-3'); and WT-1 forward primer (5'-TCT TCC GAG GCA TTC AGG AT-3') and reverse primer (5'-TGC TGA CCG GAC AAG AGT TG-3'). Relative expressions of target genes were normalized to polymerase (RNA) II (DNA directed) polypeptide A (Polr2a) with the primer pair 5'-CTC GAA ACC AGG ATG ATC TGA CTC-3' and 5'-CAC ACC CAC TTG GTC AAT GGA TAG-3', analyzed by delta-delta-CT method and are given as ratio compared to control experiments.

### Annexin V assay

Cells were incubated with TGFβ or were stretched for 24h at 37°C. Subsequently, cells were washed with warm HT buffer (137 mmol/L NaCl, 2.7 mmol/L KCl, 0.5 mmol/L MgCl_2_, 1.8 mmol/L CaCl_2_, 5 mmol/L glucose, 0.36 mmol/L NaH_2_PO_4_, 10 mmol/L HEPES, pH 7.4) and Annexin V-FLUOS (Roche Applied Science) was added following instructions of the manufacturer. DAPI was used to stain nuclei. Annexin-stained cells were visualized by confocal fluorescent microscopy (LSM Zeiss 510 meta) with the 488-nm laser line. Alternatively, Annexin V-FLUOS stained cells (3x10^5^ cells/3.5 cm well) were scraped in lysis buffer (137 mmol/L NaCl, 20 mmol/L Tris/HCl, pH 8.0, 5 mmol/L EDTA, 10% glycerol, 1% Triton X-100) and fluorescence intensity was quantified by excitation at 488 nm and emission at 520 nm by a Victor 2 fluorescent microplate reader (Perkin Elmer laboratories). 

### Rac and Rho GTPase activity assay

Active Rac-1 and RhoA were measured using G-LISA Rac-1 Activation Assay Biochem kit and G-LISA RhoA Activation Assay Biochem kit (colorimetric assay; Cytoskeleton) as instructed by the manufacturer. The signal was measured at 490 nm with a microplate reader (MRX, Dynatech Laboratories). Results were expressed as fold increase in activity of stimulated in relation to non-stimulated controls normalized to protein content. 

### Western blotting

Cells were lysed in buffer containing 50 mmol/L Tris/HCl, pH 7.5, 150 mmol/L NaCl, 10 mmol/L NaPPi, 20 mmol/L NaF, 1% Triton X-100, 2 mmol/L ortho-vanadate, 10 nmol/L okadeic acid, 40 mg/L PMSF, supplemented with protease inhibitor mix (antipain, aprotinin, leupeptin, chymostatin, pepstatin, trypsin inhibitor; 2 mg/ml each; all from AppliChem). Tissue lysates were prepared by mortaring kidneys in liquid nitrogen and dissolving the powder in the same lysis buffer. After determination of protein concentration by Bradford assay equal amounts of proteins were boiled in Laemmli buffer and separated on a 10% acrylamide SDS-PAGE gel. After transfer of proteins onto nitrocellulose membranes the following primary antibodies were used: anti-fibronectin (Sigma-Aldrich), ERK (Cell Signaling). Secondary fluorescent-coupled antibodies CF770 goat anti-rabbit IgG (Biotium) and Alexa Fluor 680 donkey anti-mouse IgG (Invitrogen) were visualized by a LICOR infrared laser scanner. Densitometry was done with the Odyssey package and results were normalized to total ERK content and data are given as mean ± SEM.

### Statistical analysis

Data are given as means ± SEM analyzed by paired or unpaired *t* test or analyses of variance (ANOVA), with and without repeated measurements, followed by Fisher´s LSD post hoc test, depending on the experimental design. Differences were considered significant at a *P* value of <0.05.

## Supporting Information

Figure S1 **GTPase inhibition improves renal blood filtration dysfunction induced by 5/6Nx.** Concentration of urea (**A**) and creatinine (**B**) in plasma 8 weeks after induction of 5/6Nx. Data are means ± SEM. *P<0.05 *vs*. corresponding sham control, # *P*<0.05 *vs*. 5/6Nx control (n=4 for urea and n=4-12 for creatinine measurement).
(TIF)

Figure S2 **GTPase inhibition attenuates 5/6Nx-induced renal fibrosis.** Tubulointerstitial damage index (**A**) and fibrosis index (**B**) in non-treated sham and 5/6Nx mice or after 8 weeks of treatment. Data represent means ± SEM. *P<0.05 *vs*. corresponding sham control (n=5-7 for sham and n=8-18 for 5/6Nx mice); # *P*<0.05 *vs*. 5/6Nx control (n=9-18).
(TIF)

Figure S3 **Podocyte characterization.** Isolated podocytes were characterized by expression of specific podocyte proteins nephrin (**A**) and WT-1 (**B**) by RT-PCR compared to mouse liver. Results are representative of 3 independent isolations and are means ± SEM.
(TIF)

## References

[1] Levey AS, Coresh J (2012) Chronic kidney disease. Lancet: 379: 165-180. doi:10.1016/S0140-6736(11)60178-5. PubMed: 21840587. 21840587

[2] Turner JM, Bauer C, Abramowitz MK, Melamed ML, Hostetter TH (2012) Treatment of chronic kidney disease. Kidney Int: 81: 351-362. doi:10.1038/ki.2011.380. PubMed: 22166846. 22166846

[3] Brenner BM (1985) Nephron adaptation to renal injury or ablation. Am J Physiol: 249: F324-F337. PubMed: 3898871. 3898871 10.1152/ajprenal.1985.249.3.F324

[4] Riser BL, Cortes P, Yee J (2000) Modelling the effects of vascular stress in mesangial cells. Curr Opin Nephrol Hypertens: 9: 43-47. doi:10.1097/00041552-200001000-00008. PubMed: 10654824. 10654824

[5] Kriz W, Elger M, Nagata M, Kretzler M, Uiker S et al. (1994) The role of podocytes in the development of glomerular sclerosis. Kidney Int Suppl: 45: S64-S72. PubMed: 8158902. 8158902

[6] Kretzler M, Koeppen-Hagemann I, Kriz W (1994) Podocyte damage is a critical step in the development of glomerulosclerosis in the uninephrectomised-desoxycorticosterone hypertensive rat. Virchows Arch: 425: 181-193. PubMed: 7952502. 7952502 10.1007/BF00230355

[7] Kerjaschki D (2001) Caught flat-footed: podocyte damage and the molecular bases of focal glomerulosclerosis. J Clin Invest: 108: 1583-1587. doi:10.1172/JCI200114629. PubMed: 11733553. 11733553 PMC201002

[8] Schiffer M, Bitzer M, Roberts IS, Kopp JB, ten DP et al. (2001) Apoptosis in podocytes induced by TGF-beta and Smad7. J Clin Invest: 108: 807-816. doi:10.1172/JCI200112367. PubMed: 11560950. 11560950 PMC200928

[9] Dessapt C, Baradez MO, Hayward A, Dei CA, Thomas SM et al. (2009) Mechanical forces and TGFbeta1 reduce podocyte adhesion through alpha3beta1 integrin downregulation. Nephrol Dial Transplant: 24: 2645-2655. doi:10.1093/ndt/gfp204. PubMed: 19420102. 19420102

[10] Durvasula RV, Petermann AT, Hiromura K, Blonski M, Pippin J et al. (2004) Activation of a local tissue angiotensin system in podocytes by mechanical strain. Kidney Int: 65: 30-39. doi:10.1111/j.1523-1755.2004.00362.x. PubMed: 14675034. 14675034

[11] Miner JH (1999) Renal basement membrane components. Kidney Int: 56: 2016-2024. doi:10.1046/j.1523-1755.1999.00785.x. PubMed: 10594777. 10594777

[12] Wilson E, Sudhir K, Ives HE (1995) Mechanical strain of rat vascular smooth muscle cells is sensed by specific extracellular matrix/integrin interactions. J Clin Invest: 96: 2364-2372. doi:10.1172/JCI118293. PubMed: 7593624. 7593624 PMC185888

[13] Ridley AJ, Schwartz MA, Burridge K, Firtel RA, Ginsberg MH et al. (2003) Cell migration: integrating signals from front to back. Science: 302: 1704-1709. doi:10.1126/science.1092053. PubMed: 14657486. 14657486

[14] Burridge K, Wennerberg K (2004) Rho and Rac take center stage. Cell: 116: 167-179. doi:10.1016/S0092-8674(04)00003-0. PubMed: 14744429. 14744429

[15] Sawada N, Li Y, Liao JK (2010) Novel aspects of the roles of Rac1 GTPase in the cardiovascular system. Curr Opin Pharmacol: 10: 116-121. doi:10.1016/j.coph.2009.11.004. PubMed: 20060361. 20060361 PMC2843793

[16] Palmer SC, Craig JC, Navaneethan SD, Tonelli M, Pellegrini F et al. (2012) Benefits and harms of statin therapy for persons with chronic kidney disease: a systematic review and meta-analysis. Ann Intern Med: 157: 263-275. doi:10.7326/0003-4819-157-4-201208210-00007. PubMed: 22910937. 22910937 PMC3955032

[17] Petermann AT, Pippin J, Durvasula R, Pichler R, Hiromura K et al. (2005) Mechanical stretch induces podocyte hypertrophy in vitro. Kidney Int: 67: 157-166. doi:10.1111/j.1523-1755.2005.00066.x. PubMed: 15610239. 15610239

[18] Hostetter TH, Rennke HG, Brenner BM (1982) The case for intrarenal hypertension in the initiation and progression of diabetic and other glomerulopathies. Am J Med: 72: 375-380. doi:10.1016/0002-9343(82)90490-9. PubMed: 7036732. 7036732

[19] Kshirsagar AV, Joy MS, Hogan SL, Falk RJ, Colindres RE (2000) Effect of ACE inhibitors in diabetic and nondiabetic chronic renal disease: a systematic overview of randomized placebo-controlled trials. Am J Kidney Dis: 35: 695-707. doi:10.1016/S0272-6386(00)70018-7. PubMed: 10739792. 10739792

[20] Amann B, Tinzmann R, Angelkort B (2003) ACE inhibitors improve diabetic nephropathy through suppression of renal MCP-1. Diabetes Care: 26: 2421-2425. doi:10.2337/diacare.26.8.2421. PubMed: 12882873. 12882873

[21] Anderson RJ, Ray CJ, Popoff MR (2000) Evidence for Rho protein regulation of renal tubular epithelial cell function. Kidney Int: 58: 1996-2006. doi:10.1111/j.1523-1755.2000.00372.x. PubMed: 11044220. 11044220

[22] Hsu HH, Hoffmann S, Endlich N, Velic A, Schwab A et al. (2008) Mechanisms of angiotensin II signaling on cytoskeleton of podocytes. J Mol Med (Berl): 86: 1379-1394. doi:10.1007/s00109-008-0399-y. PubMed: 18773185. 18773185

[23] Giehl K, Graness A, Goppelt-Struebe M (2008) The small GTPase Rac-1 is a regulator of mesangial cell morphology and thrombospondin-1 expression. Am J Physiol Renal Physiol: 294: F407-F413. PubMed: 18045834. 18045834 10.1152/ajprenal.00093.2007

[24] Shibata S, Nagase M, Yoshida S, Kawarazaki W, Kurihara H et al. (2008) Modification of mineralocorticoid receptor function by Rac1 GTPase: implication in proteinuric kidney disease. Nat Med: 14: 1370-1376. doi:10.1038/nm.1879. PubMed: 19029984. 19029984

[25] Meyer-Schwesinger C, Dehde S, von RC, Gatzemeier S, Klug P et al. (2009) Rho kinase inhibition attenuates LPS-induced renal failure in mice in part by attenuation of NF-kappaB p65 signaling. Am J Physiol Renal Physiol: 296: F1088-F1099. doi:10.1152/ajprenal.90746.2008. PubMed: 19225047. 19225047

[26] Komers R (2011) Rho kinase inhibition in diabetic nephropathy. Curr Opin Nephrol Hypertens: 20: 77-83. doi:10.1097/MNH.0b013e32834131f8. PubMed: 21076299. 21076299

[27] Peng F, Wu D, Gao B, Ingram AJ, Zhang B et al. (2008) RhoA/Rho-kinase contribute to the pathogenesis of diabetic renal disease. Diabetes: 57: 1683-1692. doi:10.2337/db07-1149. PubMed: 18356410. 18356410

[28] Komers R, Oyama TT, Beard DR, Tikellis C, Xu B et al. (2011) Rho kinase inhibition protects kidneys from diabetic nephropathy without reducing blood pressure. Kidney Int: 79: 432-442. doi:10.1038/ki.2010.428. PubMed: 20962741. 20962741

[29] Kanda T, Wakino S, Hayashi K, Homma K, Ozawa Y et al. (2003) Effect of fasudil on Rho-kinase and nephropathy in subtotally nephrectomized spontaneously hypertensive rats. Kidney Int: 64: 2009-2019. doi:10.1046/j.1523-1755.2003.00300.x. PubMed: 14633123. 14633123

[30] Olson MF (2008) Applications for ROCK kinase inhibition. Curr Opin Cell Biol: 20: 242-248. doi:10.1016/j.ceb.2008.01.002. PubMed: 18282695. 18282695 PMC2377343

[31] Löhn M, Plettenburg O, Ivashchenko Y, Kannt A, Hofmeister A et al. (2009) Pharmacological characterization of SAR407899, a novel rho-kinase inhibitor. Hypertension: 54: 676-683. doi:10.1161/HYPERTENSIONAHA.109.134353. PubMed: 19597037. 19597037

[32] Thamilselvan V, Menon M, Thamilselvan S (2012) Selective Rac1 inhibition protects renal tubular epithelial cells from oxalate-induced NADPH oxidase-mediated oxidative cell injury. Urol Res: 40: 415-423. doi:10.1007/s00240-011-0405-7. PubMed: 21814770. 21814770 PMC3694200

[33] Yao HY, Chen L, Xu C, Wang J, Chen J et al. (2011) Inhibition of Rac activity alleviates lipopolysaccharide-induced acute pulmonary injury in mice. Biochim Biophys Acta: 1810: 666-674. doi:10.1016/j.bbagen.2011.03.020. PubMed: 21511011. 21511011

[34] Binker MG, Binker-Cosen AA, Gaisano HY, Cosen-Binker LI (2008) Inhibition of Rac1 decreases the severity of pancreatitis and pancreatitis-associated lung injury in mice. Exp Physiol: 93: 1091-1103. doi:10.1113/expphysiol.2008.043141. PubMed: 18567599. 18567599

[35] Zhang QG, Wang R, Han D, Dong Y, Brann DW (2009) Role of Rac1 GTPase in JNK signaling and delayed neuronal cell death following global cerebral ischemia. Brain Res: 1265: 138-147. doi:10.1016/j.brainres.2009.01.033. PubMed: 19368836. 19368836 PMC3801190

[36] Kawarazaki W, Nagase M, Yoshida S, Takeuchi M, Ishizawa K et al. (2012) Angiotensin II- and salt-induced kidney injury through Rac1-mediated mineralocorticoid receptor activation. J Am Soc Nephrol: 23: 997-1007. doi:10.1681/ASN.2011070734. PubMed: 22440899. 22440899 PMC3358757

[37] Shibata S, Mu S, Kawarazaki H, Muraoka K, Ishizawa K et al. (2011) Rac1 GTPase in rodent kidneys is essential for salt-sensitive hypertension via a mineralocorticoid receptor-dependent pathway. J Clin Invest: 121: 3233-3243. doi:10.1172/JCI43124. PubMed: 21765214. 21765214 PMC3148723

[38] Mu Y, Gudey SK, Landström M (2012) Non-Smad signaling pathways. Cell Tissue Res: 347: 11-20. doi:10.1007/s00441-011-1201-y. PubMed: 21701805. 21701805

[39] Wilkes MC, Repellin CE, Hong M, Bracamonte M, Penheiter SG et al. (2009) Erbin and the NF2 tumor suppressor Merlin cooperatively regulate cell-type-specific activation of PAK2 by TGF-beta. Dev Cell: 16: 433-444. doi:10.1016/j.devcel.2009.01.009. PubMed: 19289088. 19289088 PMC2792748

[40] Lee HS (2012) Mechanisms and consequences of TGF-ss overexpression by podocytes in progressive podocyte disease. Cell Tissue Res: 347: 129-140. doi:10.1007/s00441-011-1169-7. PubMed: 21541658. 21541658 PMC3250617

[41] Lv Z, Hu M, Zhen J, Lin J, Wang Q et al. (2012) Rac1/PAK1 signaling promotes epithelial-mesenchymal transition of podocytes in vitro via triggering beta-catenin transcriptional activity under high glucose conditions. Int J Biochem Cell Biol: 45: 255-264. PubMed: 23153508. 23153508 10.1016/j.biocel.2012.11.003

[42] Liu YZ, Liu M, Zhang YM, Kang L, Chen PZ et al. (2012) Protective Effects of Rosuvastatin in Experimental Renal Failure Rats via Improved Endothelial Function. Biol Res Nurs. 10.1177/1099800411432630 22544519

[43] Ma LJ, Fogo AB (2003) Model of robust induction of glomerulosclerosis in mice: importance of genetic background. Kidney Int: 64: 350-355. doi:10.1046/j.1523-1755.2003.00058.x. PubMed: 12787428. 12787428

[44] Giunti S, Calkin AC, Forbes JM, Allen TJ, Thomas MC et al. (2010) The pleiotropic actions of rosuvastatin confer renal benefits in the diabetic Apo-E knockout mouse. Am J Physiol Renal Physiol: 299: F528-F535. doi:10.1152/ajpcell.00504.2009. PubMed: 20554645. 20554645

[45] Togawa A, Miyoshi J, Ishizaki H, Tanaka M, Takakura A et al. (1999) Progressive impairment of kidneys and reproductive organs in mice lacking Rho GDIalpha. Oncogene: 18: 5373-5380. doi:10.1038/sj.onc.1202921. PubMed: 10498891. 10498891

[46] Endlich N, Kress KR, Reiser J, Uttenweiler D, Kriz W et al. (2001) Podocytes respond to mechanical stress in vitro. J Am Soc Nephrol: 12: 413-422. PubMed: 11181788. 11181788 10.1681/ASN.V123413

[47] Wang YX, Martin-McNulty B, da CV, Vincelette J, Lu X et al. (2005) Fasudil, a Rho-kinase inhibitor, attenuates angiotensin II-induced abdominal aortic aneurysm in apolipoprotein E-deficient mice by inhibiting apoptosis and proteolysis. Circulation: 111: 2219-2226. doi:10.1161/01.CIR.0000163544.17221.BE. PubMed: 15851596. 15851596

[48] Wu J, Li J, Hu H, Liu P, Fang Y et al. (2012) Rho-kinase inhibitor, fasudil, prevents neuronal apoptosis via the Akt activation and PTEN inactivation in the ischemic penumbra of rat brain. Cell Mol Neurobiol: 32: 1187-1197. doi:10.1007/s10571-012-9845-z. PubMed: 22552888. 22552888 PMC11498490

[49] Jin S, Ray RM, Johnson LR (2006) Rac1 mediates intestinal epithelial cell apoptosis via JNK. Am J Physiol Gastrointest Liver Physiol: 291: G1137-G1147. doi:10.1152/ajpgi.00031.2006. PubMed: 16798728. 16798728

[50] Leblond B, Petit S, Picard V, Taverne T, Schweighoffer F (10-9-2004) COMPOUNDS AND METHODS OF TREATING CELL PROLIFERATIVE DISEASES, RETINOPATHIES AND ARTHRITIS. PCT/IB2004/000926. Patent.

[51] Désiré L, Bourdin J, Loiseau N, Peillon H, Picard V et al. (2005) RAC1 inhibition targets amyloid precursor protein processing by gamma-secretase and decreases Abeta production in vitro and in vivo. J Biol Chem: 280: 37516-37525. doi:10.1074/jbc.M507913200. PubMed: 16150730. 16150730

[52] Jung O, Jansen F, Mieth A, Barbosa-Sicard E, Pliquett RU et al. (2010) Inhibition of the soluble epoxide hydrolase promotes albuminuria in mice with progressive renal disease. PLOS ONE: 5: e11979. doi:10.1371/journal.pone.0011979. PubMed: 20694143. 20694143 PMC2915917

[53] Haas CS, Amann K, Schittny J, Blaser B, Müller U et al. (2003) Glomerular and renal vascular structural changes in alpha8 integrin-deficient mice. J Am Soc Nephrol: 14: 2288-2296. doi:10.1097/01.ASN.0000082999.46030.FE. PubMed: 12937305. 12937305

[54] Takemoto M, Asker N, Gerhardt H, Lundkvist A, Johansson BR et al. (2002) A new method for large scale isolation of kidney glomeruli from mice. Am J Pathol: 161: 799-805. doi:10.1016/S0002-9440(10)64239-3. PubMed: 12213707. 12213707 PMC1867262

