# Supplementary figures and images for: Activation of Rac-1 and RhoA Contributes to Podocyte Injury in Chronic Kidney Disease
Source: PLoS One. 2013 Nov 7;8(11):e80328. doi: 10.1371/journal.pone.0080328 (PMC3820652; doi:10.1371/journal.pone.0080328)

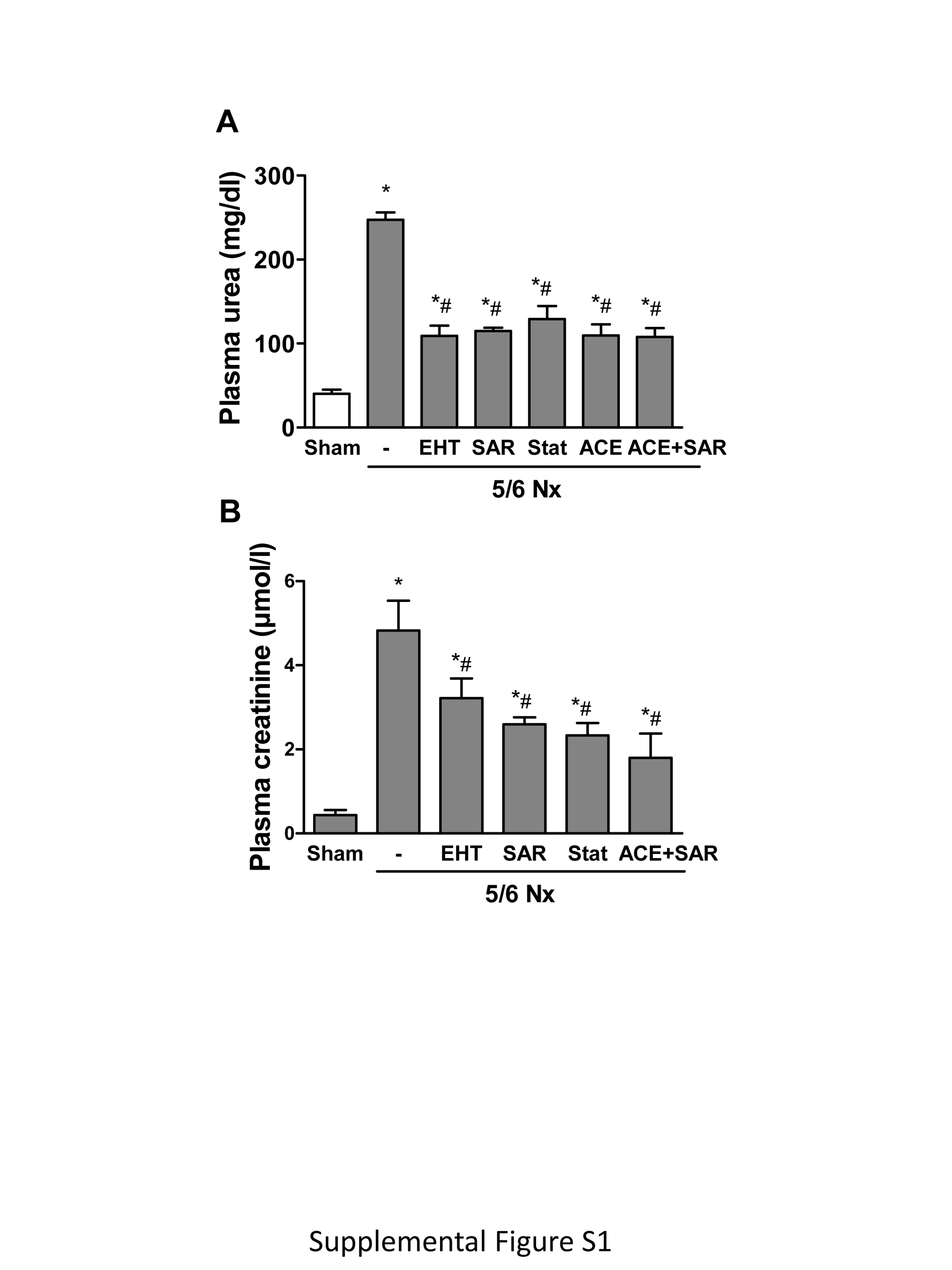

Supplement: Figure S1 — GTPase inhibition improves renal blood filtration dysfunction induced by 5/6Nx. Concentration of urea (A) and creatinine (B) in plasma 8 weeks after induction of 5/6Nx. Data are means ± SEM. *P<0.05 vs. corresponding sham control, # P<0.05 vs. 5/6Nx control (n=4 for urea and n=4-12 for creatinine measurement). (TIF) [file pone.0080328.s001.tif]

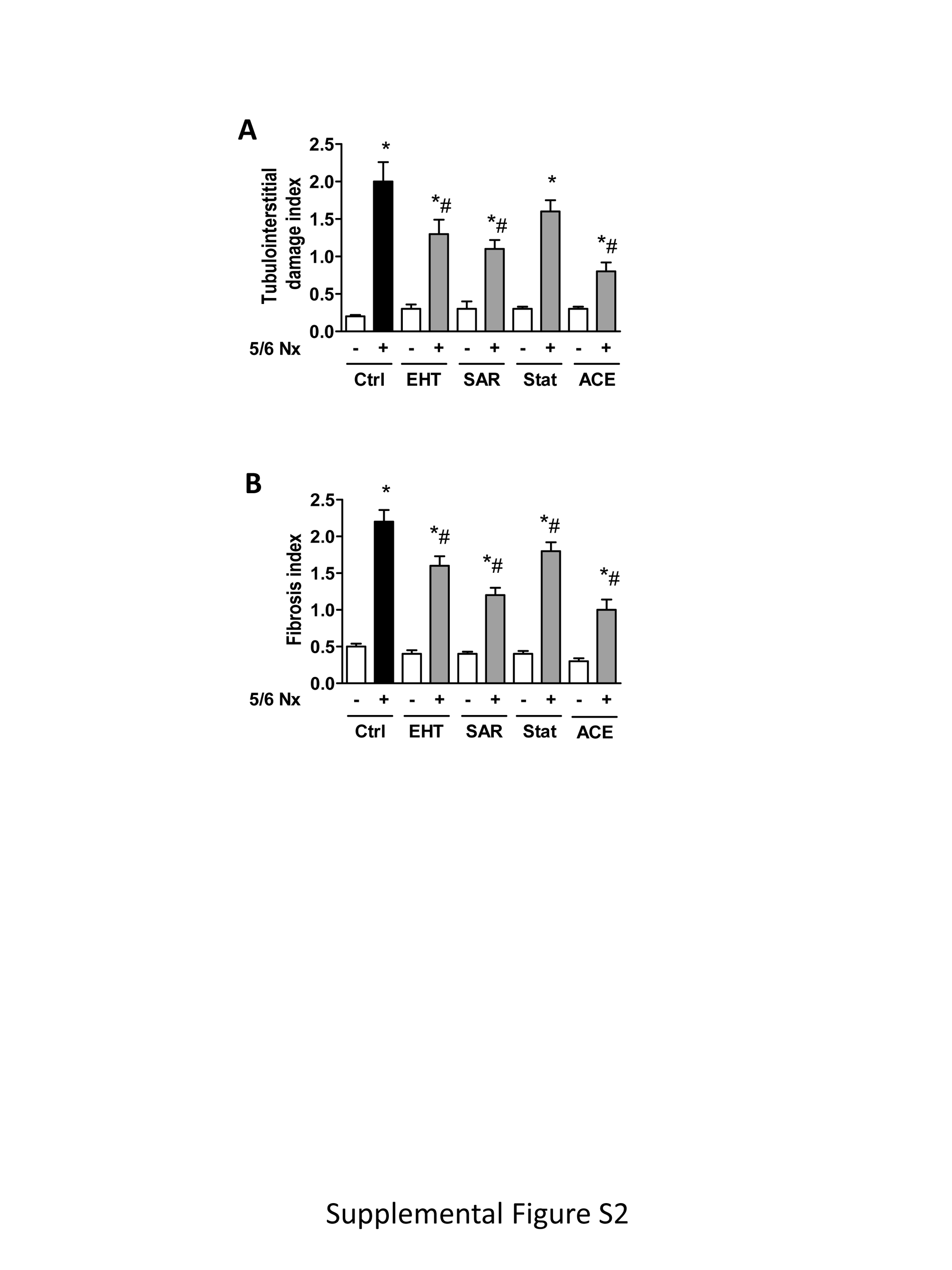

Supplement: Figure S2 — GTPase inhibition attenuates 5/6Nx-induced renal fibrosis. Tubulointerstitial damage index (A) and fibrosis index (B) in non-treated sham and 5/6Nx mice or after 8 weeks of treatment. Data represent means ± SEM. *P<0.05 vs. corresponding sham control (n=5-7 for sham and n=8-18 for 5/6Nx mice); # P<0.05 vs. 5/6Nx control (n=9-18). (TIF) [file pone.0080328.s002.tif]

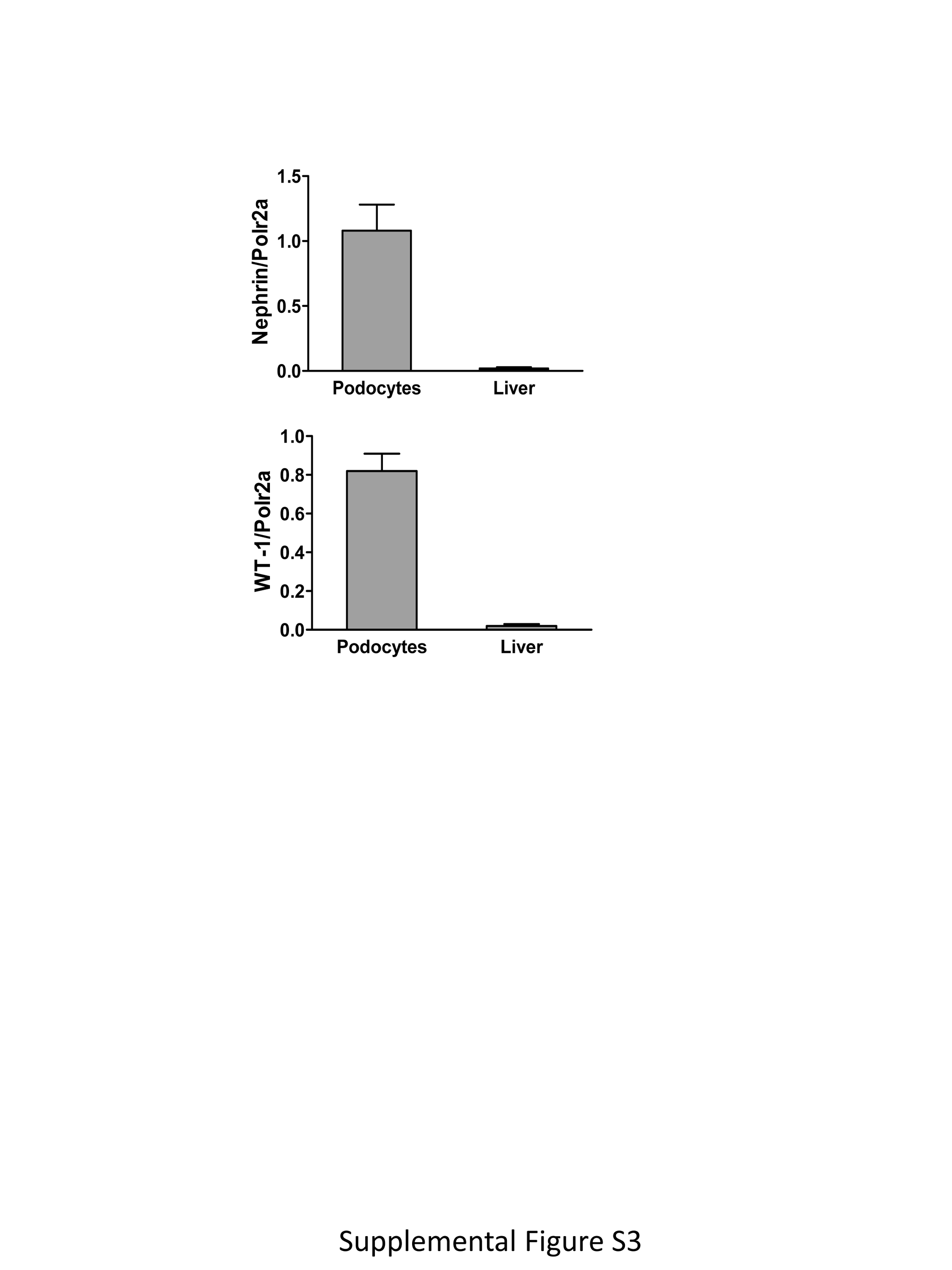

Supplement: Figure S3 — Podocyte characterization. Isolated podocytes were characterized by expression of specific podocyte proteins nephrin (A) and WT-1 (B) by RT-PCR compared to mouse liver. Results are representative of 3 independent isolations and are means ± SEM. (TIF) [file pone.0080328.s003.tif]
